# Automated Cold Embossing for the Integration of Optical Lenses onto the Surface of Acrylonitrile Butadiene Styrene (ABS) 3D-Printed Parts

**DOI:** 10.3390/polym17131745

**Published:** 2025-06-24

**Authors:** Christian A. Griffiths, Andrew Rees, Adam J. Morgan, Andrew J. Thomas

**Affiliations:** 1 Faculty of Science and Engineering, Swansea University, Swansea SA1 8EN, UK; adam.j.morgan@swansea.ac.uk; 2 Wolfson School of Mechanical, Electrical and Manufacturing Engineering, Loughborough University, Loughborough LE11 3TU, UK; a.rees@lboro.ac.uk; 3 Swansea School of Management, Swansea University, Swansea SA1 8EN, UK; a.j.thomas@swansea.ac.uk

**Keywords:** additive manufacturing, automation, cold embossing, diffractive optical lens, vapour smoothing

## Abstract

This paper presents an experimental study of a novel automated manufacturing process that integrates cold embossing to add complex features, such as micro-Fresnel lens designs, onto a 3D-printed ABS polymer component. The research demonstrates that precise control over process parameters, including embossing time (E_t_) and velocity (E_v_), is critical for successful feature replication. Gloss analysis confirmed that surface softening as a crucial prerequisite for embossing was successfully achieved using a vapour smoothing (VS) chamber that was developed and optimised for the process. High-speed automation using a 6-axis KUKA robot allowed 48 embosses to be completed in just over one minute, highlighting its efficiency over conventional hot embossing (HE) methods. Results showed that an E_t_ (0.01 s) prevented feature replication as there was insufficient time to allow for polymer flow, while an optimal E_t_ (0.1 s) produced high-quality embosses across all test segments. Additionally, this study identified that while insufficient cycle times hinder polymer flow, extended durations can lead to surface hardening, prohibiting replication. These findings pave the way for integrating Diffractive Optical Elements into 3D-printed parts, potentially enhancing precision, functionality, and productivity beyond the capabilities of standard 3D-printing processes.

## 1. Introduction

New and existing technologies on the market have increased the necessity of highly functional optical elements. High surface quality Diffractive Optical Elements (DOEs) offer an alternative to traditional refractive optics due to their flexibility, reduced thickness, and consequently light weight, as well as relatively low material consumption [1]. DOEs are typically used in equipment for applications in telecommunication, imaging, lithography, biomedical devices, optical sensors, and cameras [2,3,4,5,6,7].

Injection moulding (IM) is the main replication method for the mass manufacturing of polymer components. However, when replicating high-aspect-ratio micro- and nanoscale features over large areas, HE is utilised [8,9]. HE has flexibility whereby it can be used for both small-scale and mass production and has proven to be a cost-effective technology for polymer lens manufacturing [10,11]. In addition, HE offers several benefits, such as lower manufacturing costs due to the ability to utilise a single-stage mould for mass production. HE also yields excellent results with regards to precision [12,13].

The process of HE requires that the temperature of a polymer substrate be raised above the polymer glass transition temperature (T_g_). Then the mould insert moves towards the substrate. During contact between the mould insert and the substrate, an embossing force (F_e_) is applied [14]. Next, the cooling stage of the process begins at which time the polymer and mould are kept in contact above T_g_, and a slow cooling cycle is applied to reduce internal stresses (a requirement for optical functionality) [15]. The final stage is part demoulding whereby the mould and polymer are separated from each other by opening the tool. Tool and substrate adhesion is a very delicate stage of the process as the demoulding forces can inadvertently damage the replicated features [16].

To enable plastic deformation and material flow, the following process factors are considered: melting point of the polymer (T_m_), embossing temperature (T_e_), embossing pressure (P_e_), and embossing time (E_t_). For the manufacturing of polymer devices, microfluidic optimum replication results differ. In a study by Chen et al., an optimum process yield was achieved at an embossing temperature of 130 °C and embossing time of 5 min [17]. Yong he et al. found that optimum replication fidelity was achieved when utilising a T_e_ of 150 °C, T_e_ of 30 min, and P_e_ of 300 N [18]. Cogun et al. concluded that optimum replication results were observed at T_e_ of 115 °C, P_e_ of 10 KN, and T_e_ of 8 min [19]. In the research by Deshmukh et al., high accuracy was achieved with a T_e_ of 135 °C, P_e_ of 30 kg/cm^2^, and E_t_ of 180 s. In addition, the same study also observed that embossing temperature has a crucial impact on the replication accuracy, and it contributes 70.52% of the overall process accuracy [12].

Melentiev and Lubineau [20] found that three common characteristics of the HE method are as follows: (1) The majority of the replicated structures have sub-mm sizes, typically 100–400 µm channels, dimples, and pyramids [21,22,23,24]. HE at the lower end of the micro-scale (1–10 µm) is exceptionally rare. (2) The majority of the hot embossed parts have coin-sized areas, e.g., microfluidics [25,26], micro-lenses [27,28,29], and micro-electromechanical systems (MEMS) [30,31]. Many of the studies have been performed on poly (methyl methacrylate) (PMMA) [31,32,33,34,35,36,37].

For the manufacturing of lenses, the advantages of HE can be witnessed in the low material flow rate, which prevents internal stresses and facilitates the replication of delicate microstructures [38]. For mass-market, the process is suitable for the manufacturing of diffractive structured surfaces such as holograms [39]. Temperature control challenges within the process have resulted in the evolution of the process whereby ultrasonic hot embossing has been developed. Within this process configuration, a sonotrode and pressure are applied to soften the plastic to allow flow into the micropattern of the mould. Further advantages of the US hot embossing process include its ability to be conducted at room temperature [40].

Rooney et al. created a robust method to manufacture and characterise the optical performance of transparent 3D-printed, high-quality bulk optics using a consumer-grade printer and commercially available resin [41]. Contact lenses have been manufactured using the direct laser printing (DLP) technique, and nanopatterns were textured on the surface of the contact lenses with the help of direct laser interference patterning (DLIP) [42]. Stereolithography (SLA) 3D-printing of transparent resin lens was performed where the surface roughness, light transmittance, and morphologies of SLA-fabricated lens were examined by atomic force microscopy, UV spectrophotometer, and scanning electron microscopy [43]. SLA printing of Fresnel lenses exhibited over 90% of optical transmittance and the printed lenses confirmed the feasibility of the 3D printing process for the fabrication of optical devices [44]. Muntaha et al. reported a comparison between a commercially available straight cylindrical lens and custom 3D-printed, curved cylindrical lenses [45]. Ma Q et al. discussed additive manufacturing (AM) processes like Fused Deposition Modelling (FDM), SLA, material jetting, and binder jetting, highlighting their applications in radar/sensing, communications, EMI shielding, and electromagnetic absorption. They identified that the challenges include optimising material properties, intricate structural design, manufacturing precision, and performance validation [46].

Acrylonitrile Butadiene Styrene (ABS) is a widely used commercial polymer for demanding engineering applications [47,48,49]. However, for the HE of ABS, only limited research has been conducted [20]. In a study by Melentiev and Lubineau, it identified that ABS is made up of strongly entangled polymeric chains, and thus has a high activation energy [20]. Further studies also concluded that when using ABS, high activation energy is required by molecules to move against the frictional forces, and thus polymer flow is two to three times higher than those of the other polymers [50]. To initiate flow in moulds, the applied energy must be greater than the internal flow resistance of the polymer. To achieve flow, higher T_e_ can be used to reduce the resistance of flow into a mould cavity. Typically, temperatures higher than the T_g_ are required [51,52,53,54]. However, increases in temperature result in excessive thermal shrinkage when cooling, which can distort the replicas [13]. To address this challenge, Melentiev and Lubineau used a viscous embossing strategy for surface micropatterning of ABS products. This process used a T_e_ far above T_g_ and demoulded shortly after, with the polymer in the viscous state. The absence of cooling resulted in the replication of 1 µm structures over a large surface area. This proposed strategy exhibits several advantages such as highly precise and more productive replication, which are not observed in the conventional HE of ABS [20].

Many researchers have dedicated their efforts to improve the surface finish and dimensional accuracy of Fused Deposition Modelling (FDM) prototypes. The research has concluded that the surface finish of FDM parts can be greatly enhanced through exposure to specific chemicals. One notable advancement in this area is Stratasys, Inc.’s vapour smoothing (VS) process. This method creates a controlled environment for vaporising specialised chemicals, allowing hot chemical vapours to react with the upper surface of FDM parts. VS has shown success, particularly with FDM ABS parts, utilising Acetone vapour treatment to enhance the surface finish [55,56,57,58,59,60]. The chemical vapours enhance the surface finish of ABS replicas by softening the external layer. Acetone breaks the secondary bonds between ABS polymer chains, enabling the chains to slide past one another and settle into more stable positions. Specifically, on the surface of the ABS part, acetone erodes material from the upper surface, prompting a re-flow process that effectively fills the air gaps created during the layer-by-layer fabrication of the part [61]. However, there is no research that demonstrates a cold embossing (temperature between 18 and 21 °C) process for ABS 3D-printed parts after the VS stage.

Numerous studies shown the potential for significant improvement in the surface finish. Within this process there is a post-processing opportunity to further modify the surface using micropattern tooling. By utilising polymer re-flow in the HE processes, it is possible to modify the surface of ABS parts at room temperature, thereby negating the T_e_. Using a developed VS post-processing chamber and integrating it into an automation cell with a 6-axis robot, this research will demonstrate a production line for micro-texturing of an ABS substrate with a Fresnel lens design. The imprinting process will be automated, and then the quality of the lens replication will be compared to the master tool pattern.

The paper is organised as follows: Section 2 discusses the experimental setup, as well as the test apparatus and methodology adopted to investigate the capability of the replication process. Next, in Section 3, the design of experiments for conducting the research is discussed together with the approach adopted for analysing the results. The experimental results are presented and the relationship between VS process parameters and replication quality is analysed. Section 4 discusses the results of the experiment design. Finally, in Section 5, the main conclusions from the conducted study and recommendations for improved automation of the VS cold embossing process is presented.

## 2. Experimental Materials and Methods

**Figure 1:**
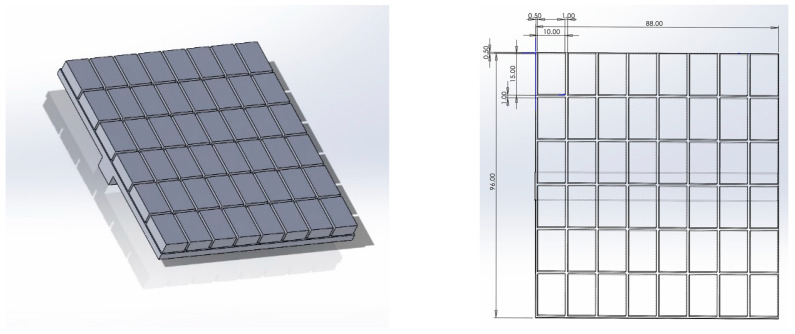
Test part design.

To investigate the replication performance of the cold embossing process, two hypotheses are considered. The first hypothesis (H1) will establish if the developed VS conditions for ABS are correctly set for the cold embossing process. For this, three identical 3D-printed parts (Figure 1) are produced, with one then subjected to VS. Gloss analysis is performed on both parts to test H1. The second hypothesis (H2) will establish process factors that influence the replication quality of the cold embossing process using a high-precision Fresnel lens design. First, a mass-production injection moulding tool insert will be integrated to a specialised end effector, then a fully automated cold embossing process will be performed on a test part. Finally, the parts will be inspected for replication fidelity using a Dino-lite edge digital microscope AnMo Electronics Corporation New Taipei City Taiwan and a Alicona Infinite Focus microscope (Bruker Alicona, Graz, Austria).

**Figure 2:**
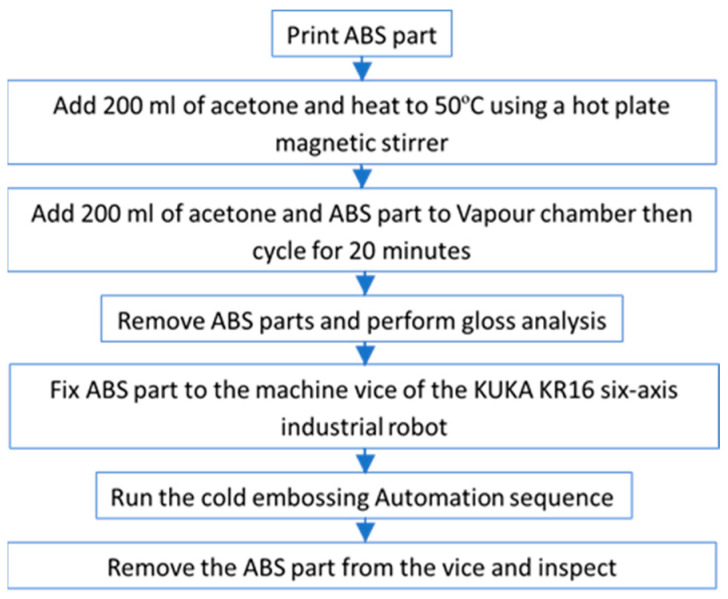
Flow chart of the process.

Pre-trials have shown that the cold embossing process can texture the surface of ABS polymer parts. For H2, three main experiments are to be performed (Table 1). For each experiment, the automation controls for embossing time (E_t_) and embossing velocity (E_v_) are varied and the difference between the experiments is measured in cycle time. Forty-eight embossing tests are performed for each experiment and metrology is used to measure the replication fidelity population means. A flow chart describes the complete process (Figure 2). The following section will describe the research setup of the cold embossing process.

### 2.1. Test Part

#### 2.1.1. Emboss Part Design and 3D Printing

An 88 mm × 96 mm × 5 mm test part is used for automated embossing (Figure 2). The part is segmented (1 mm gap) for each of the emboss positions; there are six rows of eight so the test part design can accommodate a total of fort eight embosses. The printer used for manufacturing the parts is a Bambu Lab X1E Carbon (Bambu Lab, Shenzhen, China). This model is a high-resolution 3D printer known for its advanced features and professional capabilities. It excels in fast printing speeds while producing intricate designs and prototypes in fine detail. Automatic calibration is used, ensuring optimal print quality and minimising manual adjustments while supporting and multi-material printing. Compatible with various 3D printing software, the X1E offers flexibility in design and print preparation, with software features for optimising settings and reducing environmental impact through energy-efficient operation and eco-friendly filament support. The slicer used throughout was Bambu Studio v1.9.4, and the filament used was white Acrylonitrile Butadiene Styrene (ABS) supplied by RS Components (Northamptonshire, UK). A total of eight parts were produced to establish optimum settings (Table 1).

#### 2.1.2. Vapour Chamber

**Figure 3:**
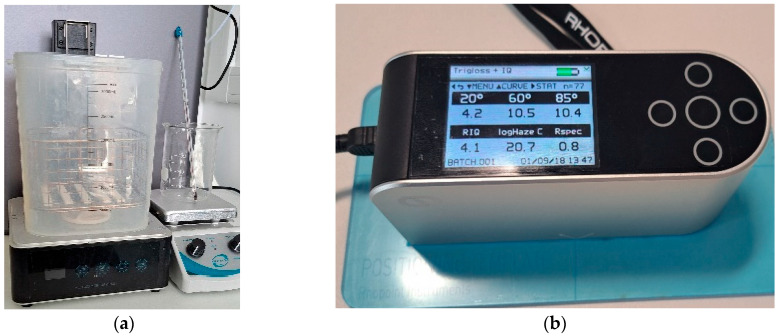
(**a**) Vapour station. (**b**) Rhopoint Q Gloss meter.

To vapour smooth the parts after printing, an ELEGOO Wash and Cure Station Mercury Plus 2.0 (Elegoo Inc., Shenzhen, China) has been repurposed (Figure 3a). The station is designed to work with isopropyl alcohol (IPA) and other suitable cleaning solutions, and the wash functionality of the system is utilised for acetone. The cure station allows for a volume of 131 mm × 90 mm × 220 mm and has a customisable cycle. The rotating impeller at the bottom of the wash and cure station container creates a swirling motion that provides a 360-degree cleaning action. This action stirs the acetone, preventing it from being stationary and aiding vapour flow within the station. The selected acetone (Table 2) is pre-treated before being added to the station. Specifically, 200 mL of acetone is heated to 50 °C using a hot plate magnetic stirrer, ensuring a consistent temperature. The acetone is then transferred to the tank, followed by the ten test parts pre-positioned within the wash basket. Once the protective lid is in place, the VS cycle is run for 20 min. After the cycle is complete, the parts are removed from the station and left to solidify prior to gloss inspection.

#### 2.1.3. Gloss Analysis

Gloss is the visual sensation associated with the brightness of direct light reflected from a surface. Surfaces with high reflectance are classified as glossy, while less reflective surfaces are categorised as semi-gloss or matte. Gloss meters quantify this effect by measuring the light reflection from a sample at defined angles. The Gloss Unit (GU) is defined in international standards such as ISO 2813 [62] and ASTM D523 [63]. It is determined by the amount of light reflected from a glass standard with a known refractive index. In this research, a Rhopoint Q Gloss meter (St Leonards-on-Sea, East Sussex, UK) is used (Figure 3b) and a standard diode array optical configuration of 20° is used to measure the distribution of the reflected light. The Rhopoint IQ Gloss Meter have an associated uncertainty of ±0.4 GU, as defined by the ISO 17025 [64] calibration standard. Before conduction the cold embossing automation process it is necessary to establish the efficacy of the VS process. To achieve this, measurements will be taken for both non-VS parts and VS parts, and the results will be presented in Section 3.

### 2.2. Embossing Stamper

#### 2.2.1. Fresnel Lens Stamper

**Figure 4:**
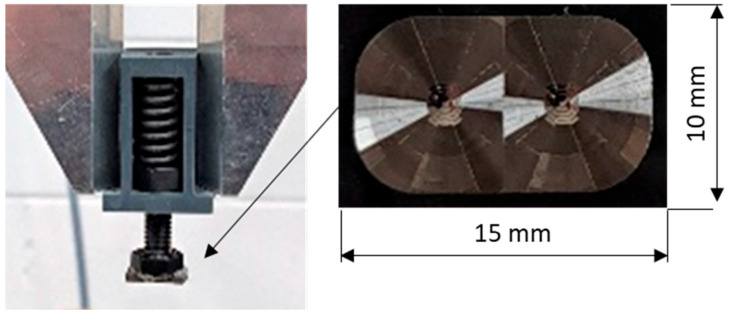
Stamper assembly.

A mobile phone optical Fresnel lens is a thin and light product that is integrated to a mobile phone with limited space. The lenses are made from cost-effective lens-grade polymers such as polycarbonate. The design utilises concentric rings where each ring acts as an individual prism element that bends light to a focal point. Used for photography and videography, the lens can magnify via zoom and enhance the phone’s display. When used with the phone’s light source, the lens can project the phone’s screen onto a larger surface, allowing for advanced functionality. A mobile phone optical Fresnel lens is a thin, lightweight component designed for integration with mobile devices, where space is critical. Constructed from cost-effective, lens-grade polymers such as polycarbonate, these lenses employ a design based on concentric rings. Each ring functions as an individual prism element, bending light towards a focal point. This innovative structure allows the Fresnel lens to achieve significant optical performance while maintaining a minimal form factor. In photography and videography, the Fresnel lens enhances the phone’s capabilities by providing zoom magnification, thereby improving the quality and detail of captured images. Additionally, it can be used to amplify the phone’s display, making on-screen content easier to view. When paired with the phone’s light source, the Fresnel lens also enables the projection of the phone’s screen onto larger surfaces and expands the potential applications of mobile devices. To make these lenses, a mass-production injection moulding master tool insert is used (Figure 4). The tool is 10 × 15 × 1 mm, while the dual Fresnel feature covers an area of ≈104 mm^2^ and incorporates >50 concentric rings for mobile phone applications. The stamper is bonded to a m8 bolt that is housed in a polymer case. To prevent damage to the lens stamper, the housing accommodates a spring-loaded system that allows for 10 mm of travel. The whole stamper assembly is held in a Zimmer GPP5010NC gripper system (Zimmer Group GmbH, Rheinau, Germany).

#### 2.2.2. Stamper Force Test

**Figure 5:**
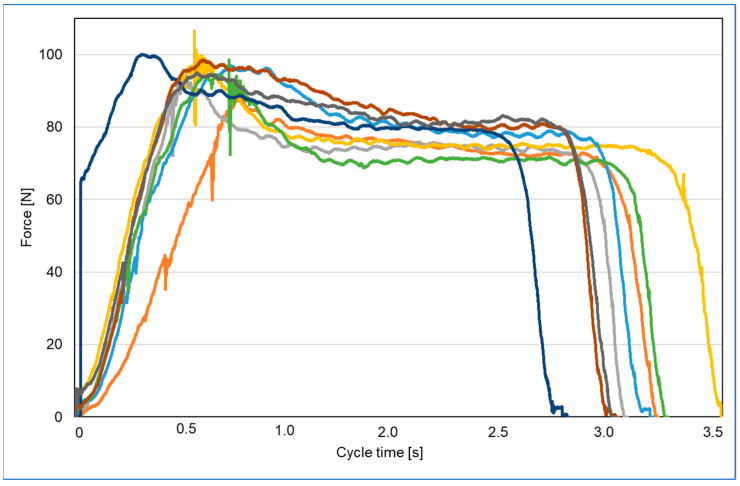
Embossing force calibration.

In this research, the stamper is programmed to automatically stamp the test part. To establish the F_e_ delivered, a force platform (model number 9260AA, Kistler Instruments AG, Winterthur, Switzerland) was used to collect force data prior to conducting the main experiments. The platform’s vertical range was set to its minimum, 0–500 N (i.e., 0–250 N per corner transducer). The analogue signal from the force platform was sampled at a frequency of 2000 Hz through a 16-bit analogue-to-digital converter (ADC) using Kistler’s data acquisition system (DAQ) (Type 5691A, Kistler Instruments Ltd.) and Bioware software 5.3 (Type 2812A; Kistler Instruments Ltd.). The force platform was factory-calibrated and, before testing, underwent satisfactory calibration checks using masses that were traceable to national standards. A stamping cycle of up to 3.5 s was selected and eight tests were performed (Figure 5). The results show that the stamper assembly will deliver a mean F of 97.3 N to the test part (Table 3).

### 2.3. Automation

#### 2.3.1. KUKA Robot

**Figure 6:**
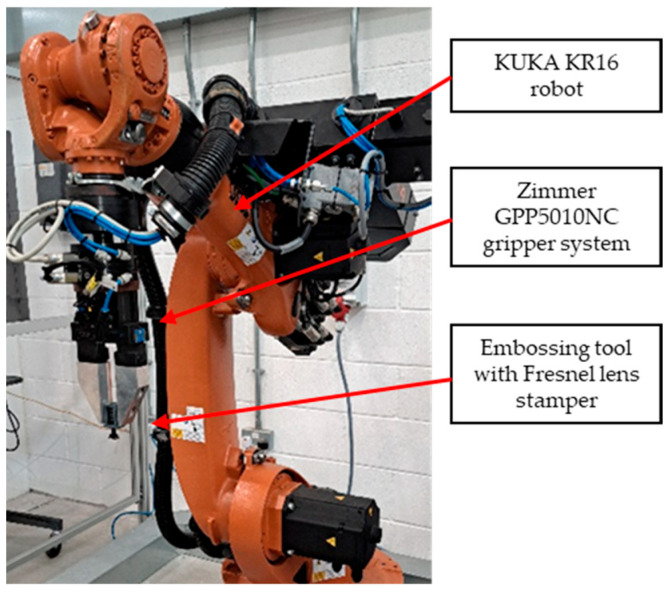
Kuka KR 16 robot and stamper tool.

This study was conducted using a KUKA KR16 six-axis industrial robot (Augsburg, Germany) (Figure 6). The robot has a positional repeat ability accuracy of ±0.04 mm, payload of 16 kg, and a maximum reach of 1610 mm. A machine tool vice is used to hold the test parts, and a KUKA BASE frame is set and calibrated to the vice to ensure the robot is accurately positioned in relation to the test part. The Zimmer GPP5010NC gripper system used to hold the embossing tool is integrated to the KR16 and the KUKA TOOL calibration procedure is performed to set the stamper to the selected BASE.

#### 2.3.2. Sequence

All the assembly positions were programmed using KUKA KRL language. The incremental programming function of the robot is used, and the programme sequence follows a raster scanning approach where the robot moves to each emboss site on the test part (Figure 2) horizontally left-to-right. Then after completing all of the embossing steps, it moves back to the left on the next line down. This is completed for each of the 48 embossing positions until it reached the final position at right-hand side of the bottom row.

For the embossing stage of the programme, the tool is positioned 1 mm above the test part. Once the stamper is in contact with the part, further movement in the Z-direction of 10 mm is made. This ensures that the spring-loaded stamper applies a controlled Fe (>90 N) to the test part surface. Three emboss velocities of 0.1, 0.01, and 0.001 mm/s will be used; Table 4 shows the performed experiments.

## 3. Results

### 3.1. Gloss Results

**Figure 7:**
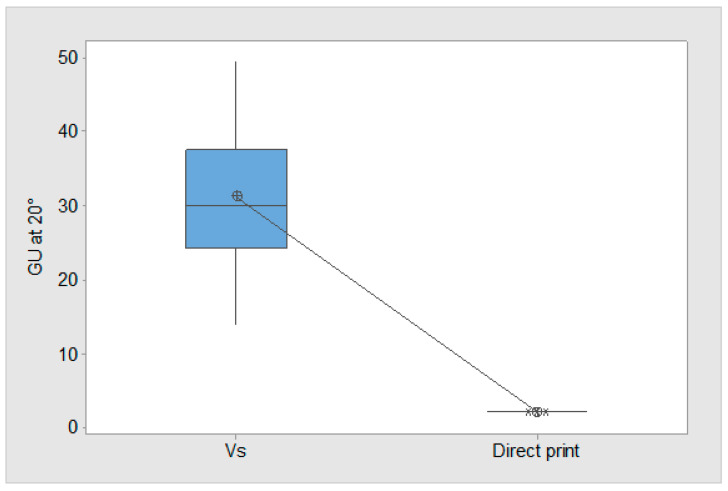
Boxplot of GU results for VS and non-VS parts.

To establish the correct conditions for the cold embossing process, two identical parts were 3D-printed (Figure 2) and one was subjected to VS. Gloss analysis was then performed on both parts. The pooled average of all the 169 measurements can be used to test the hypothesis (H1) that the developed VS process has a significant influence on gloss on the test parts. The GU result is a direct indication that surface softening has occurred, which is a pre-condition for cold embossing. The Two-Sample T-Test result shows that the *p*-value is below 0.05 (Table 5), confirming that there is a significant difference between the means, and H1 is correct. The mean gloss increase of the VS-processed part is 1461.5% at 20°, thus providing evidence for the efficacy of the VS process (Figure 7). Figure 7 shows a significant increase in gloss for VS 3D-printed parts compared to directly printed parts. The VS process raised the GU from near zero to a median of around 30 GU, with a wider range of values. This confirms that VS effectively enhances surface finish by increasing gloss, making it a viable method for improving the aesthetics and surface quality of 3D-printed components. The standard error of the mean (SE Mean) result that estimates the variability between fitted means shows that there is a wider distribution of VS parts. The GU results highlight variation at the test part surface. However, inspection showed that softening occurred throughout the top layer, thus allowing the part to progress to the cold embossing process.

### 3.2. Replication

The results of the cold embossing experiments (see Table 4) show that for Experiment 1, no replication was observed; this result indicates that E_t_ is a critical factor for replication. Specifically, the speed process factor in this experiment did not allow the polymer to flow into the lens cavities effectively.

**Figure 8:**
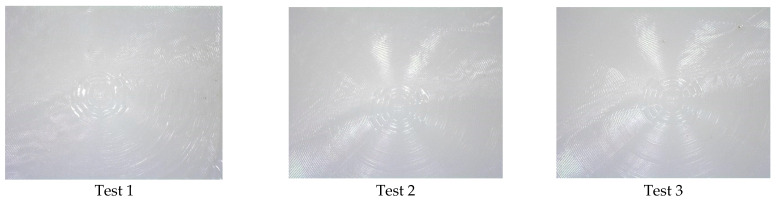

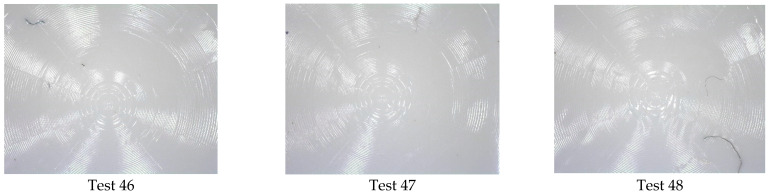
Experiment 2 test results.

**Figure 9:**
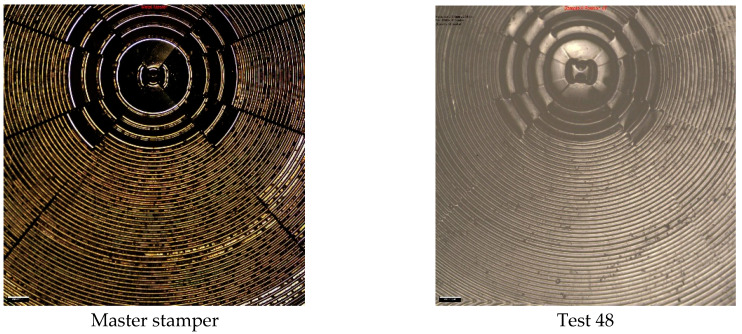
Experiment 2 replication images for the master tool and 48th test.

Experiment 2 is presented in Figure 8, where the images for the first three replications (Test 1–3) and the last three replications are shown (test 46–48). The images show that replication of the lenses is present on the polymer surface (some contamination is observed). Further replication analysis using Alicona Infinite Focus variation was used to compare the master tool against the 48th cold emboss test performed in Experiment 2. The image shown in Figure 9 provides further evidence that the process can replicate complex geometrical surfaces.

**Figure 10:**
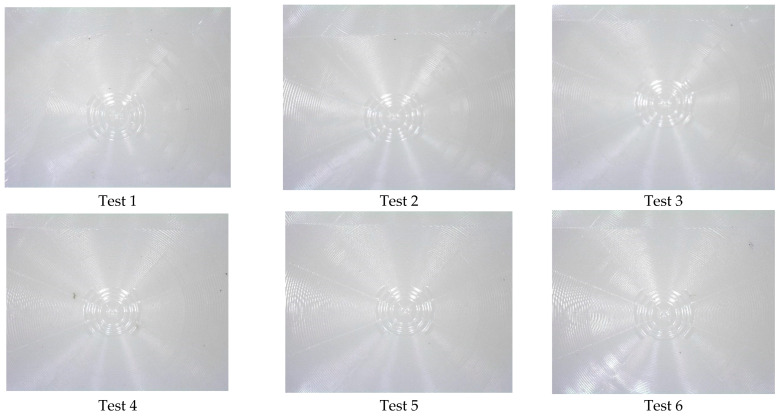

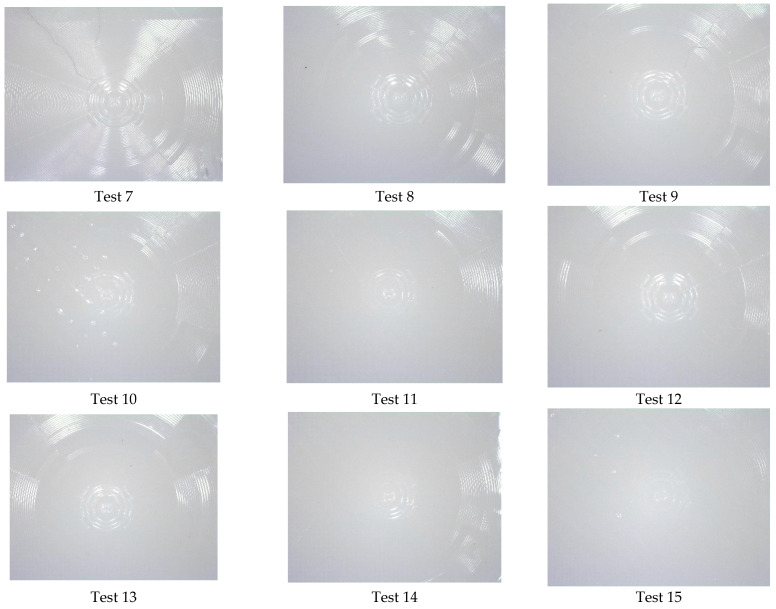
Experiment 3 test results.

**Figure 11:**
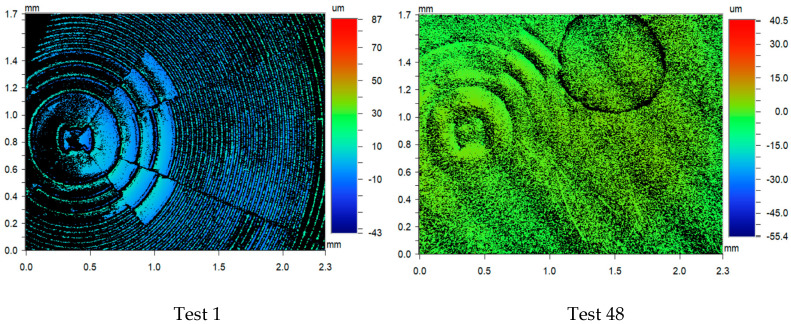
Experiment 3 replication images for the 1st and 48th tests.

The control parameters for Experiment 3 allowed for replication, but fidelity declined after the 7th replicate (Figure 10). By the 15th test, only a faint replication of the lens was achieved. Further imaging (Figure 11) showed that test 1 was successful and that a faint replication exists after the 48th test.

**Figure 12:**
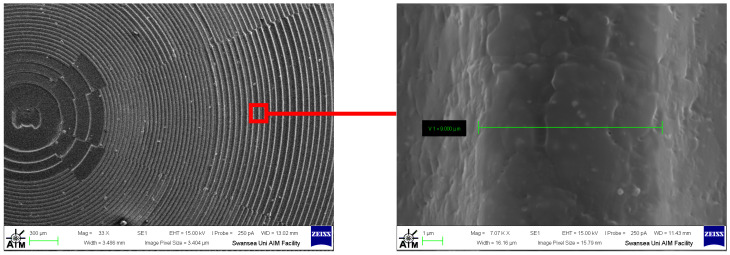
Experiment 1 replication image using SEM.

To complete the characterisation, the polymer test part was coated with a thin metal layer of platinum using sputter coating using an Agar (Cressington) HR Sputter coater (Agar Scientific, Rotheram, UK) to enhance its surface conductivity for scanning electron microscopy (SEM) analysis. A film thickness monitor terminated the coating when approximately 10 nm of platinum was applied. This was verified by a calibration check post-process. The sputtering process ensured uniform coverage, minimising charging effects during imaging. Subsequent SEM inspection of Experiment 1 using a Zeiss Evo LS25 SEM (Jena, Germany) enabled high-resolution visualisation of the component’s surface morphology, revealing fine structural details of the Fresnel lens and topographical ring features (Figure 12). The image shows that the process is capable of micro-scale replication; in particular, the lens ring thickness of 9 µm is replicated. This level of replication is normally associated with µ-injection moulding and time-consuming HE.

This result highlights the influence of E_t_ on the process, as the softening of the test piece diminishes over time, affecting the polymer’s ability to mould to the lens features. The cycle time plays a crucial role; a duration of 454 ms for an emboss is insufficient for adequate polymer flow to replicate the master, while an increased time of 9364 results in the polymer hardening, thereby hindering replication. This finding is significant as it demonstrates that cold embossing can be utilised to incorporate complex features into 3D-printed parts. Moreover, optimising the cycle time can enhance productivity. This research provides a pathway for improving the precision of embossed features while maintaining the structural integrity of 3D-printed components.

## 4. Discussion

The results of this experiment demonstrate that vapour-smoothed (VS) cold embossing is a viable manufacturing method for imprinting micro-scale details, such as Fresnel lens designs, onto 3D-printed ABS components. The replication of these details was achieved with a high degree of accuracy, where E_t_ and E_v_ parameters played a crucial role in ensuring fine-detail reproduction. A successful VS process was developed to enable cold embossing, and a set of E_t_ values was established for optimal embossing. Shorter E_t_ times hindered polymer flow, while excessively long times reduced replication quality.

These findings confirm the initial hypothesis that cold embossing can be effectively achieved through this method, providing a viable alternative to HE, which requires significantly higher temperatures and processing times. This alternative approach offers potential benefits, including lower manufacturing costs and reduced energy consumption. As a novel technique, this research could serve as a foundation for further studies on its feasibility in industrial applications.

However, several limitations were identified. Firstly, the VS method employed in this experiment was adapted using repurposed equipment, introducing variability in the process that could affect results. The GU measurements in Figure 6 show a range of values obtained during testing, suggesting that a more tightly controlled process could improve accuracy and consistency. Additionally, when transferring VS parts from the vapour chamber to the embossing setup, timings were not measured, introducing the possibility that the ABS polymer began solidifying before embossing. Furthermore, the displacement of polymer during embossing was not measured, which could lead to inaccuracies in smaller components due to material overflow.

Future work should investigate these factors further, including refining the VS process for greater consistency and assessing whether polymer overflow differs between cold and HE. Additionally, integrating robotic automation could enhance accuracy by ensuring a consistent transition time between VS and embossing, improving reproducibility and precision.

## 5. Conclusions

This paper reports an experimental study on a novel manufacturing process for adding complex features to 3D-printed components. Automation and cold embossing have been used to imprint lens features onto the surface of ABS polymer parts. This scientific finding underscores the delicate balance required in cold embossing processes, where precise control over parameters like E_t_ and cycle time is essential for successful replication. The following conclusions can be made based on the reported research:

- The first hypothesis (H1) established that the developed VS conditions are correctly set for the cold embossing process. Gloss analysis is performed on the parts to test H1. The pooled average of all 169 measurements show that the VS has a significant influence on gloss on the test parts. The GU result evidences that surface softening has occurred, which is a pre-condition for cold embossing.
- Conventional HE of polymers is a time-consuming process. In this research the second hypothesis (H2) establishes that a high-speed automation process with correctly controlled factors for cold embossing can imprint Fresnel lens design features onto 3D-printed ABS parts. It was demonstrated that 48 embosses can be performed in just over one minute.
- Replication quality is dependent on embossing time (E_t_) and embossing velocity (E_v_). A low E_t_ of 0.01 s resulted in no observed replication of the lens features. In contrast, an increase in E_t_ to 0.1 s yielded optimal results, with all tests across 48 segments of the test piece successfully embossed.
- This study reveals that polymer flow is hindered by insufficient cycle times, but excessive durations lead to surface-hardening of the polymer, obstructing effective replication.

The insights from this research pave the way for advancements in integrating complex features, such as Diffractive Optical Elements, into 3D-printed parts. Automation enhances the potential for both precision and productivity, enabling the production of 3D-printed products with functionalities that exceed standard printing capabilities. However, for broad adoption of this novel method across various 3D-printed products and ABS-based applications, future research must address several challenges associated with traditional HE of polymers. These challenges include feature characterisation, replication fidelity of nanoscale structures, adhesion and demoulding forces between the stamper and component, air evacuation, incomplete cavity filling, and precise alignment between the component and stamper.

## Figures and Tables

**Table 1:** Bambu Studio Bambu Lab X1E print setting.

Parameter	Setting
Initial Layer Height	0.2 mm
Layer Height	0.1 mm and 0.3 mm
Nozzle Diameter	0.4 mm
Initial Line Width	0.5 mm
Line Width	0.42 mm
Outer Wall	0.42 mm
Inner Wall	0.45
Top Surface	0.42
Seam Alignment	Back
Wall Line Count	2
Top Layers	5
Top Surface Pattern	Monotonic Line
Bottom Layers	5
Infill Pattern	Rectilinear
Infill Density	100%
Supports	Off
Build Plate Adhesion	None
Printing Temperature	260 °C
Build Plate Temperature	90 °C

**Table 2:** Acetone properties.

Material group	100,043
CAS Reg No	67-64-1
Density @20 °C	0.789–0.792
Water (% Mass)	0.3%
Refractive index @20 °C	1.359
Assay (% Mass)	99.8
Acidity (As Acebic Acid)—(% Mass)	Max 0.002

**Table 3:** Force results.

Mean 97.3 N	SE Mean 1.82	StDev 5.16
95% confidence mean for F measurements is between 93.0 and 101.7 N		
95% confidence for median F is between 94.0 and 100.5 N		
95% confidence interval for StDev of 3.14 to 10.5		

**Table 4:** Experiment array.

Experiment	E_t_ (s)	E_v_ (mm/s)	Total Cycle Time (ms)	Average Cycle Time/Test (ms)
1	1.0	0.1	21,780	454
2	0.1	0.01	60,228	1254
3	0.01	0.001	449,472	9364

**Table 5:** Two-Sample T-Test gloss results.

Sample	Mean	StDev	SE Mean
VS	31.23	8.44	0.69
Non-VS	2.085	0.036	0.008
T-Value	DF	*p*-Value	
42.13	148	0.000	

## Data Availability

The original contributions presented in this study are included in the article. Further inquiries can be directed to the corresponding author.

## Abbreviations

The following abbreviations are used in this manuscript:

DOEs	Diffractive Optical Elements
HE	Hot Embossing
CE	Cold Embossing
ABS	Acrylonitrile Butadiene Styrene
FDM	Fused Deposition Modelling
VS	Vapour Smoothing
IPA	Isopropyl Alcohol
GU	Gloss Unit
ADC	Analogue to Digital Converter
DAQ	Data Acquisition System
IM	Injection Moulding
T_g_	Glass Transition Temperature
F_e_	Embossing Force
T_m_	Embossing Temperature
P_e_	Embossing Pressure
E_t_	Embossing Time
E_v_	Embossing velocity
MEMS	Micro-Electro Mechanical Systems
PMMA	Poly Methyl Meth Acrylate
DLP	Digital Light Processing
DLIP	Direct Laser Interference Patterning
SLA	Stereolithography
UV	Ultra Violet
AM	Additive Manufacturing
